# Roles of endogenous retroviral elements in the establishment and maintenance of imprinted gene expression

**DOI:** 10.3389/fcell.2024.1369751

**Published:** 2024-03-05

**Authors:** Sherry Fang, Kai-Wei Chang, Louis Lefebvre

**Affiliations:** Department of Medical Genetics, Life Sciences Institute, University of British Columbia, Vancouver, BC, Canada

**Keywords:** genomic imprinting, DNA methylation, H3K27me3, endogenous retroviral elements, developmental epigenetics

## Abstract

DNA methylation (DNAme) has long been recognized as a host defense mechanism, both in the restriction modification systems of prokaryotes as well as in the transcriptional silencing of repetitive elements in mammals. When DNAme was shown to be implicated as a key epigenetic mechanism in the regulation of imprinted genes in mammals, a parallel with host defense mechanisms was drawn, suggesting perhaps a common evolutionary origin. Here we review recent work related to this hypothesis on two different aspects of the developmental imprinting cycle in mammals that has revealed unexpected roles for long terminal repeat (LTR) retroelements in imprinting, both canonical and noncanonical. These two different forms of genomic imprinting depend on different epigenetic marks inherited from the mature gametes, DNAme and histone H3 lysine 27 trimethylation (H3K27me3), respectively. DNAme establishment in the maternal germline is guided by transcription during oocyte growth. Specific families of LTRs, evading silencing mechanisms, have been implicated in this process for specific imprinted genes. In noncanonical imprinting, maternally inherited histone marks play transient roles in transcriptional silencing during preimplantation development. These marks are ultimately translated into DNAme, notably over LTR elements, for the maintenance of silencing of the maternal alleles in the extraembryonic trophoblast lineage. Therefore, LTR retroelements play important roles in both establishment and maintenance of different epigenetic pathways leading to imprinted expression during development. Because such elements are mobile and highly polymorphic among different species, they can be coopted for the evolution of new species-specific imprinted genes.

## 1 Genomic imprinting and host defense mechanisms

The first mouse imprinted genes, *H19, Igf2,* and *Igf2r*, were identified in 1991 (Barlow et al., 1991; Bartolomei et al., 1991; DeChiara et al., 1991). The imprinting of *Snrpn* was demonstrated the following year (Cattanach et al., 1992; Leff et al., 1992), and in 1993 the first reports presenting evidence supporting a role for DNAme in the imprinting mechanism were published (Bartolomei et al., 1993; Brandeis et al., 1993; Ferguson-Smith et al., 1993; Li et al., 1993). It was already recognized at the time that the DNAme machinery exploited in mammals was derived from bacterial immune systems that had been adapted for the transcriptional repression of repetitive sequences (Bestor, 1990). This led the late Denise Barlow to propose that genomic imprinting had evolved from host defense mechanisms, by co-opting DNAme-based functions in the parent-of-origin-specific silencing of imprinted genes (Barlow, 1993). Several different predictions of the model proposed have been confirmed, as recently reviewed (Ondičová et al., 2020). A related aspect of this model, which is the focus of this review, addresses the roles played by endogenous repetitive elements themselves in the regulation of imprinted gene expression. Here, we review recent evidence suggesting that LTR elements have been co-opted for both germline establishment and somatic maintenance of imprinted gene expression in early development.

## 2 Retrotransposons and canonical imprint establishment

### 2.1 DNA methylation imprints

Early studies on diploid biparental gynogenetic and androgenetic embryos suggested that genomic imprinting is established during gametogenesis (McGrath and Solter, 1984; Surani et al., 1984). Although the epigenetic mechanisms involved were not known at the time, DNAme was later shown to represent an important epigenetic mark, directly inherited from the mature gametes, and regulating imprinted gene expression. The monoallelic expression of canonical imprinted genes in somatic cells is maintained by differential DNAme marks (Tucci et al., 2019) established *de novo* during male or female gametogenesis by the sex-specific action of the DNA methyltransferase DNMT3A (Kaneda et al., 2004), and its co-factor DNMT3L (Bourc’his et al., 2001; Hata et al., 2002; Arima et al., 2006). Large fractions of the genome are differentially methylated between eggs and sperm, but unlike most of the differences, the gametic DNAme marks at imprinted genes survive the wave of demethylation occurring during pre-implantation stages. This survival of imprints requires the maintenance DNA methyltransferase DNMT1 (Li et al., 1993; Hirasawa et al., 2008), its partner UHRF1 (Sharif et al., 2007), and the DNAme-dependent DNA-binding factors ZFP57 and ZFP445. These KRAB zinc-finger proteins specifically bind the methylated allele of imprinted genes and protect it from demethylation during preimplantation stages via their recruitment of KAP1/TRIM28 and SETDB1. This histone methyltransferase establishes a H3K9me3 mark over the DNAme-marked region for preferential recruitment of DNMT1 via UHRF1, which recognizes H3K9me3 via its tandem Tudor domain and plant homeodomain (Li et al., 2008; Strogantsev and Ferguson-Smith, 2012; Takahashi et al., 2019; Janssen and Lorincz, 2021). In somatic cells, sequences carrying these DNAme imprints are detected as Differentially Methylated Regions (methylated on a single allele) of gametic origin (gDMR). Imprinted gDMRs are thought to be responsible for all canonical imprinted gene expression observed in embryonic and adult cells. Only 24 gDMRs have been identified in the mouse, 21 methylated in the oocyte and 3 in sperm (Proudhon et al., 2012; Bogutz et al., 2019). Both oocyte and sperm DNAme play essential roles in imprinting, but whereas most of the paternal DNAme is lost after fertilization, a portion of oocyte-derived 5-methylcytosine (5mC) survives the passive demethylation occurring during preimplantation (Smallwood et al., 2011). Although the function of most of this inherited maternal DNAme is still unknown, some of these maternal marks were shown to be required for silencing genes detrimental for placental development, the first demonstration of a role for maternal DNAme unrelated to genomic imprinting (Branco et al., 2016). Recent surveys suggest that the human genome contains more gDMRs (Zink et al., 2018; Akbari et al., 2022), with several maternally-inherited marks maintained only in the placenta (Court et al., 2014; Hamada et al., 2016; Hanna et al., 2016; Sanchez-Delgado et al., 2016).

### 2.2 Imprint establishment during oogenesis: a transcription-guided process

The analysis of the DNAme profile of gametes at single-base resolution using whole-genome bisulphite sequencing (WGBS) revealed that there is nothing fundamentally unique about *de novo* establishment of imprinted gDMR. Rather, these sequences acquire DNAme as part of global mechanisms methylating the mouse sperm and oocyte genomes at >80% and ∼40% levels, respectively (Kobayashi et al., 2012). Mature gametes are also methylated at very different levels in human, with average levels of DNAme of ∼75% and ∼54% for sperm and egg, respectively (Okae et al., 2014a). In the mouse, whereas paternal gDMRs are DNA methylated in prospermatogonia from E14.5 to birth (Davis et al., 2000; Li et al., 2004), elegant embryological experiments showed that the establishment of functional maternal imprints occurs during the phase of oocyte growth taking place in postnatal ovaries (Kono et al., 1996; Obata et al., 1998; Obata and Kono, 2002). Accordingly, the genome of primary non-growing oocytes (NGO, from P1-P5 females), and of fully-grown, germinal vesicle stage oocytes (FGO or GVO, from mature females), show a drastic difference in average genomic DNAme levels, from 2% to 40%, including at several CpG islands (CGIs) (Shirane et al., 2013). The process of *de novo* DNAme therefore occurs postnatally in females, in non-dividing oocytes, and was shown to require DNMT3A and its cofactor DNMT3L, but not DNMT3B or the maintenance DNMT1 enzyme (Smallwood et al., 2011; Kobayashi et al., 2012; Shirane et al., 2013).

By comparing the DNA methylome and transcriptome of oocytes, as determined by RNA-seq, a direct correlation was observed between gene transcription and gene body DNAme (Smallwood et al., 2011; Kobayashi et al., 2012; Veselovska et al., 2015). Whereas promoter regions of active genes are hypomethylated (<15% 5 mC), their transcribed regions acquire 60%–90% DNAme, starting ∼2 kb downstream of their oocyte-specific transcription start site (TSS). Strikingly, transcribed regions account for 85%–90% of the methylome of FGOs, including DNAme at all imprinted maternal gDMRs (Veselovska et al., 2015). Pioneering work from the group of Gavin Kelsey showed that oocyte transcription across the gDMR region was required for DNAme establishment at the maternal *Gnasxl/Nespas* gDMR and that most imprinted maternal gDMRs are indeed covered by an oocyte transcript initiating at an upstream promoter (Chotalia et al., 2009). Following this work, a role for oocyte transcription in *de novo* DNAme at the gDMRs of the paternally expressed genes *Snrpn, Plagl1,* and *Kcnq1ot1* was demonstrated directly in mouse mutants in which inserted transcription termination sequences prevent oocyte transcripts from extending across the DMR region (Smith et al., 2011; Veselovska et al., 2015; Singh et al., 2017). Although DNAme blocks are not as well defined in human oocytes, a strong correlation was also noted between methylated and transcribed regions, suggesting that the link between *de novo* methylation and transcription is conserved (Okae et al., 2014a). Interestingly, other examples of transcription-coupled acquisition of DNAme at imprinted promoters emerged from the analysis of retrogenes, inserted within a host gene expressed in oocytes (Cowley and Oakey, 2010). Because of their location, the promoter of these inserted retrogenes is covered by a transcript in oocytes and acquires a maternal gDMR, leading to silencing of the maternal allele and expression from the paternal allele of the retrogene in the progeny (Wood et al., 2007).

The mechanism whereby transcribed regions acquire DNAme in oocytes was shown to be guided by both negative and positive cross-talks with specific histone post-translational modifications. Unmethylated promoter CGIs are usually marked by H3K4me3 (Mikkelsen et al., 2007) and this mark has an inhibitory effect on the action of the DNMT3A-DNMT3L complex (Guo et al., 2015), protecting these CpG-rich sequences from *de novo* DNA methylation (Ooi et al., 2007). This implies that intragenic CpG-rich regions covered by an oocyte transcript would be refractory to *de novo* DNA methylation unless methylation marks at H3K4 are previously removed from those regions. Consistent with this prediction, CGIs acquiring DNAme during oocyte growth are devoid of or lose H3K4me2/3 marks in preparation for *de novo* DNA methylation, and the H3K4 lysine demethylase KDM1B plays a dominant role in the removal of these refractory marks (Ciccone et al., 2009; Stewart et al., 2015). Interestingly, a mutant version of DNMT3A carrying two point mutations within the ADD domain, which interfere with the binding of DNMT3A to H3K4me0 *in vitro*, was recently shown to lead to dwarfism and female infertility. The global DNAme of oocytes from homozygous females is severely affected (at only 17.6%, compared to 35.9% for wild-type oocytes), leading to stochastic loss of maternal imprinted methylation, abnormal expression of different imprinted genes in the progeny, with variations between individual embryos, several of which die before mid-gestation (Uehara et al., 2023).

The recruitment of the *de novo* DNA methyltransferases DNMT3A and 3B to transcribed regions is mediated by their PWWP domain, a reader for H3K36me2/3 marks (Dhayalan et al., 2010). As originally demonstrated in somatic cells, transcribed regions acquire an H3K36me3 domain via the recruitment of the histone methyltransferase SETD2, which is in a complex with the elongating RNA polymerase II (Yoh et al., 2008). The SETD2-deposited H3K36me3 marks then recruit DNMT3B to those regions via its PWWP domain, leading to the establishment of a DNAme block over transcribed regions in ESCs (Baubec et al., 2015; Neri et al., 2017). A similar recruitment mechanism is conserved during *de novo* DNA methylation in the germline, although different approaches are exploited to establish the H3K36me-marked domains in male and female gametes. In oocytes, both SETD2-deposited H3K36me3 marks over transcribed regions as well as H3K36me2, presumably deposited by the nuclear receptor-binding SET-domain proteins NSD1 or NSD2, are implicated in establishing the maternal methylome via recruitment of the DNMT3A-DNMT3L complex (Xu et al., 2019; Yano et al., 2022). In the male germline, where the genome is more than 80% methylated (Kobayashi et al., 2012), the recruitment of DNMT3A is mediated by NSD1-deposited H3K36me2 marks, which cover broad regions of the genome (Shirane et al., 2020).

Despite this simple model implicating direct DNMT3A-H3K36me2/3 interactions, some results on mouse mutants carrying specific mutations in the DNMT3A PWWP reader domain may suggest that additional mechanisms are also at play during *de novo* DNA methylation. Two point mutations within the PWWP domain abrogating the binding of DNMT3A to H3K36me2/3 *in vitro* have been modeled in mouse. Both of these alleles, D239A and W236R, lead to dominant growth retardation phenotypes characterized by abnormal gain of DNAme at H3K27me3-marked regions. Surprisingly, gene bodies where H3K36me3 is deposited were unaffected (Heyn et al., 2019; Sendžikaitė et al., 2019). Similar observations were also made in mutant oocytes expressing only the D239A variant, in which H3K36me2/3-marked regions still acquired DNAme (Kibe et al., 2021). Although those results may suggest the existence of an alternative recruitment mechanism for the DNMT3A/3L complex, the authors also raise the possibility of residual binding of the D239A mutant PWWP domain to H3K36me2/3 *in vivo*, or a compensation via interactions between DNMT3A and DNMT3B, which also features an H3K36me2/3-binding PWWP domain (Kibe et al., 2021). The resolution of these alternative scenarios will require the direct analysis of DNMT3A D239A binding specificity *in vivo* by ChIP-seq and studies involving the simultaneous deletion of *Dnmt3b* in oocytes.

### 2.3 LTR elements expression in oocytes

Long-terminal-repeat retrotransposons (LTRs), also known as endogenous retroviruses (ERVs), are highly variable in mammalian genomes and constitute ∼10% and ∼9% of the mouse and human genomes, respectively (Chinwalla et al., 2002). Several families of transposable elements, mostly young LTRs, can promote transcription initiation and act as TSS during oocyte growth in both mouse and human (Peaston et al., 2004; Veselovska et al., 2015; Franke et al., 2017; Hendrickson et al., 2017). Therefore, although LTRs are usually silenced by epigenetic mechanisms implicating DNAme or repressive histone marks such as H3K9me3 (Liu et al., 2014), some of these elements, notably younger LTRs, evade these mechanisms and are active as promoter elements in growing oocytes. Some of these LTR-initiated transcripts are intergenic or antisense to known genes, but others act as oocyte-specific alternative promoters for annotated genes, forming chimeric transcripts with annotated downstream exons. This enormous potential of LTR elements to shape the oocyte transcriptome is conserved in mammals and has been documented by oocyte RNA-seq in several species, such as mouse, rat, hamster, human and cow (Franke et al., 2017; Hendrickson et al., 2017; Brind’Amour et al., 2018). Interestingly, oocytes utilize a paralogue of the general transcription factor TATA binding protein (TBP), called TBPL2 (also known as TRF3 or TBP2), for transcription initiation during oocyte growth (Gazdag et al., 2009). TBPL2 was shown to play an important role in oocyte transcription, including at LTR promoters, notably at those featuring a TATA-like motif (Yu et al., 2020). It will be interesting to document how the DNA methylome and imprinting are affected in *Tbpl2* ^*−/−*^ oocytes.

Given the high level of expression from specific LTR promoters in oocytes and the observation that transcribed regions are *de novo* methylated, a significant fraction of the oocyte methylome originates from transcription initiating in active LTR promoters (Brind’Amour et al., 2018). A comparative description of such an impact of LTR-initiated transcripts on the DNA methylome of mouse, rat, and human oocytes showed that transcriptionally active LTRs are responsible for wide differences in DNAme patterns in oocytes of different species (Brind’Amour et al., 2018). Note that as for non-repetitive oocyte promoters, the active LTRs themselves are not DNA methylated in oocytes and overlap with a peak of H3K4me3 active promoter mark. As for single-copy promoters, they also lead to the deposition of an H3K36me3 domain over the transcribed region and the subsequent formation of a block of DNAme starting ∼2 kb downstream of the TSS provided by the LTR element (Brind’Amour et al., 2018).

### 2.4 Evidence for LTR-guided imprint establishment in oocytes

Since DNAme blocks acquired in oocytes and maintained during preimplantation stages play a critical role in imprinting, the work on LTR-driven transcription and DNAme in oocytes raised the following questions: Do some of the DNAme marks acquired in oocytes as a consequence of transcription from LTRs act as imprinted gDMRs allowing only paternal allele-specific expression of the downstream gene in the progeny? Has this mechanism contributed to the evolution of species-specific imprinted genes?

**FIGURE 1:**
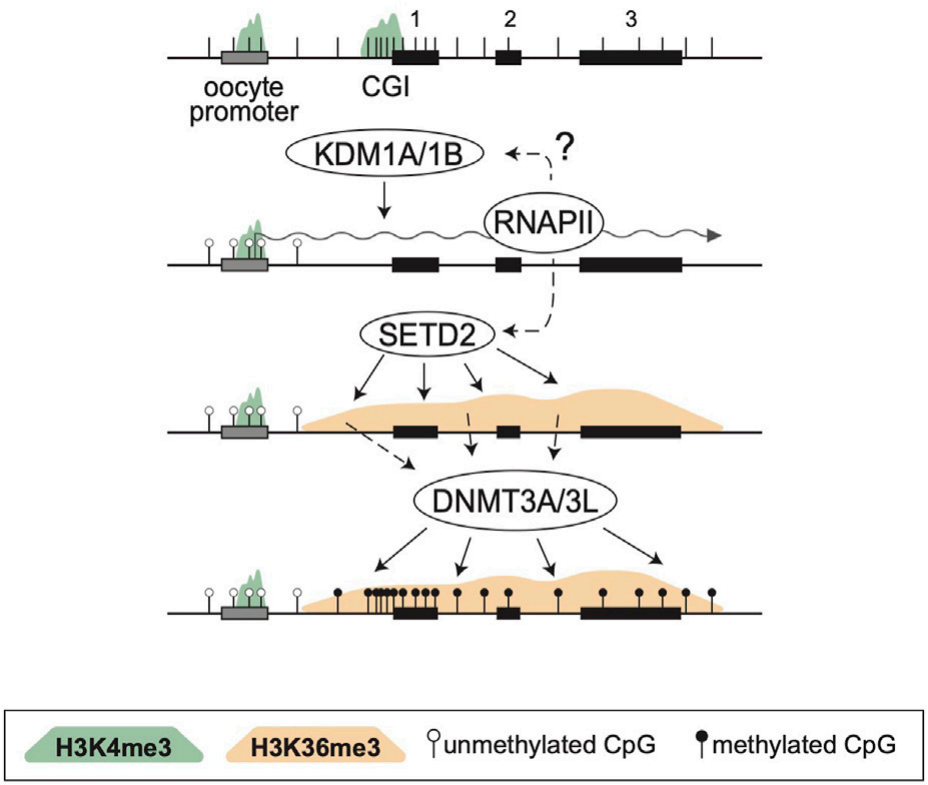
*De novo* DNA methylation during oocyte growth. Structure of a 3-exon gene is presented at the top, showing exons (black rectangles), a CGI promoter, the positions of CpG dinucleotides (vertical bars) and an upstream oocyte promoter (grey). In oocytes both promoters are marked with H3K4me3 (green shade) but KDM1A/1B, perhaps in association with RNAPII, remove this mark at the somatic CGI promoter. Simultaneously, SETD2 deposits H3K36me3 (orange shade) over the entire transcribed region. This mark is read by the PWWP domain of DNMT3A, which together with DNMT3L, methylates the transcribed region, including the CGI promoter.

**FIGURE 2:**
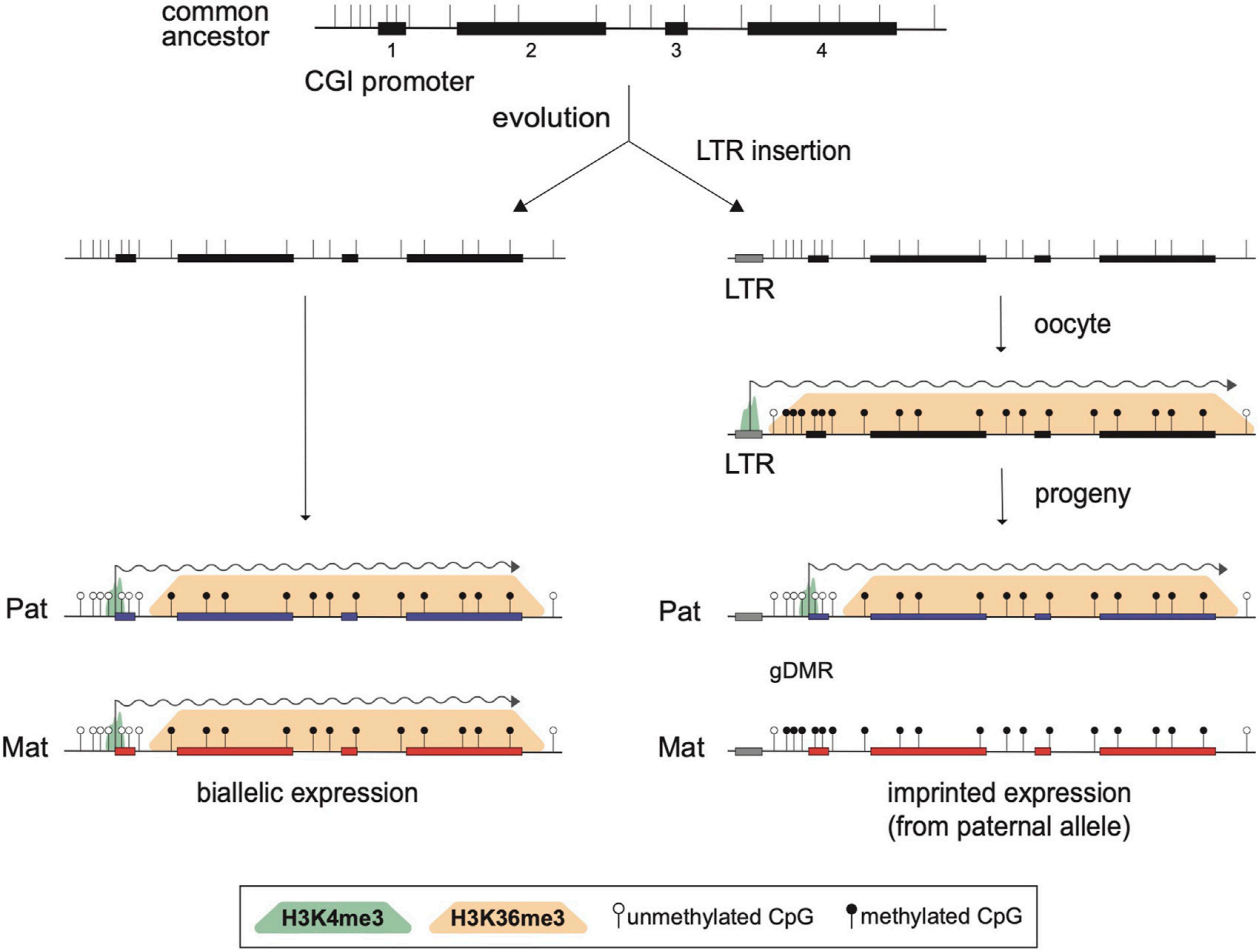
Model for the acquisition of imprinted expression via the insertion of an LTR element. Structure of a biallelically expressed ancestral gene is presented at the top, showing four exons (black rectangles), the positions of CpG dinucleotides (vertical bars), and a CpG island (CGI) promoter overlapping exon 1. Following evolution, two scenarios are considered. On the left, the locus is unchanged and the gene is expressed from both alleles in the progeny, as shown by biallelic active H3K4me3 marks (green shade) at the promoter and active transcription (wiggly arrow). One the right, a *de novo* retrotransposition event leading to the insertion of a solo LTR upstream of exon 1 is represented. The inserted LTR remains transcriptionally active in oocytes and induces the formation of blocks of H3K36me3 (orange shade) and DNAme (black lollipops) over the transcribed region. Consequently, the CGI promoter remains DNA methylated on the maternal allele in the progeny and the gene becomes a paternally expressed imprinted gene.

By analyzing known imprinted gDMRs established in mouse (21 gDMRs) and human (125 gDMRs) oocytes, 21 examples of methylated regions covered by oocyte transcripts initiated within an LTR element were identified, 4 in the mouse, and 17 in human (Bogutz et al., 2019). Based on a 2018 survey, the mouse and human genomes were found to contain approximately 260 and 228 imprinted genes, respectively, with 63 shared in both species (Tucci et al., 2019). From these figures, it follows that ∼1.5% and ∼7.5% of imprinted genes are regulated by oocyte promoters in mouse and human, respectively. Data from mouse oocytes show that transcription initiation from these LTRs, marked by H3K4me3, lead to downstream blocks of H3K36me3 and DNAme deposition over the transcribed region, covering the site of the associated gDMR (Figure 1). Interestingly, none of these are the 15 maternal gDMRs shared between those two species. Moreover, for the 4 mouse gDMRs, the oocyte transcripts all initiate within LTR families specific to rodents, and 12 of the 17 human gDMRs are covered by transcripts initiating from LTRs of primate-specific families, 9 of which appear conserved in chimpanzee. Whereas most of this data is correlational, CRISPR-Cas9 mediated deletions of the LTR elements acting as upstream oocyte promoters at the mouse *Impact* and *Slc38a4* imprinted genes confirmed the importance of these elements in species-specific maternal imprints. For both LTR knockouts, DNAme is lost at the gDMR of those genes in oocytes of homozygous knockout females and imprinting is lost in the progeny, with biallelic transcription of each gene (Bogutz et al., 2019). Together, the analysis presented in this study highlights a previously unappreciated role for LTR elements of endogenous retroviruses: by acting as promoters in oocytes, some of these elements can induce DNAme at a downstream CpG-rich promoter that is otherwise kept unmethylated in sperm, and can therefore lead to the formation of a new paternally-expressed imprinted gene, assuming maintenance of this maternal DNAme mark post-fertilization (Figure 2). As mentioned above, the survival of DNAme marks at gDMRs during preimplantation development relies on the binding of ZFP57 and ZFP445 to methylated TGCCGC motifs. Most of the canonical imprinted genes regulated by oocyte-specific LTR promoters contain at least one such binding site (Table 1). For the 17 human genes, ZFP57 binding has been observed by ChIP-seq at the *HTR5A* and *CLDN23* CGI promoters, which maintain their imprinted DNAme mark in many epiblast-derived tissues (Bogutz et al., 2019). For 14 of these genes, imprinted DNAme at the CGI has only been observed in the placenta, so ZFP57/445 binding would be expected to only be observed during preimplantation development and in extra-embryonic cells.

**TABLE 1:** Canonical imprinted genes regulated by an oocyte LTR promoter.

Imprinted gene		Oocyte LTR promoter	CGI coodinates return (mm10/hg19)	DNAme maintenance	ZFP57 peak^a^	TGCCGC motif^b^
Mouse	*AK008011/Gm5790*	RMER19B	chr13:47010854-47011096	tissue-specific	yes^c^	4
	*Cdh15*	MTD	chr8:122864938-122865178	tissue-specific	yes^c^	2
	*Slc38a4*	MT2A	chr15:97054449-97054857	ubiquitous	N	2
	*Impact*	MTC	chr18:12973304-12973796	ubiquitous	yes	5
Human	*DNAH7*	MLT1A0	chr2:196933311-196933665	placental	no	0
	*MCCC1*	LTR12C	chr3:182816772-182817455	placental	no	1
	*BANK1*	LTR12E	chr4:102711830-102712199	placental	no	1
	*RHOBTB3*	LTR12C	chr5:95066877-95067812	placental	no	5
	*COL26A1*	HERVH	chr7:101005900-101007443	placental	no	4
	*SCIN*	THE1 C	chr7:12610166-12610834	placental	no	1
	*AGBL3*	MER51E	chr7:134671120-134671750	placental	no	0
	*SVOPL*	THE1D	chr7:138348963-138349444	tissue-specific	no	1
	*HTR5A*	MSTA	chr7:154862681-154863245	tissue-specific	yes	3
	*HECW1*	LTR12C	chr7:43152021-43153340	placental	no	2
	*CLDN23*	LTR12C	chr8:8559132-8560867	tissue-specific	yes	3
	*GL/S3*	MER50	chr9:4297818-4300182	placental	no	2
	*ZC3H12C*	MLT1A1	chr11:109963241-109964677	placental	no	2
	*ST8SIA1*	LTR53	chr12:22486836-22488666	placental	no	0
	*SORD*	LTR12F	chr15:45315202-45315543	placental	no	1
	*ZFP90*	MER50	chr16:68572892-68573740	placental	no	1
	*ZNF396*	MSTA	chr18:32956765-32957406	placental	no	2

^a^ ChIP-seq ZFP57 peak from ReMap Atlas of Regulatory Regions in UCSC, genome browser.

^b^ Includes motifs within and close to the CGI.

^c^ Not detected in two studies in mouse ESCs (Strogantsev et al., 2015; Anvar et al., 2016).

## 3 Retrotransposons and noncanonical imprint maintenance

### 3.1 Evidence for DNAme-independent imprinting

Although DNAme-based canonical imprinting provided an elegant mechanism to explain most imprinting effects, some observations suggested the existence of a parallel epigenetic pathway leading to parent-of-origin effects of gametic origin. For instance, a few cases of isolated imprinted genes lacking a gDMR were reported, such as the paternally expressed genes *Sfmbt2* and *Gab1* (Wang et al., 2011; Okae et al., 2012). In the placentae of cloned mice obtained by somatic cell nuclear transfer, using cumulus or Sertoli cells as nuclear donors, those same two genes, together with *Slc38a4*, were also shown to be consistently expressed from both alleles (Okae et al., 2014b). Furthermore, these three imprinted genes maintain at least some imprinted expression in the embryonic progeny of *Dnmt3l* or conditional *Dnmt3a/3b* null females, which fail to *de novo* methylate their oocyte genome (Okae et al., 2012; 2014b). Since the imprinted expression of canonical gDMR-regulated genes was faithfully maintained in most cloned mice, the authors concluded their study of *Sfmbt2, Gab1,* and *Slc38a4* with this insightful prediction: “It is likely that an imprinting mark[s] other than DNA methylation may be required for the establishment of imprinting of these genes” (Okae et al., 2014b).

Similarly, research in the field of imprinted X chromosome inactivation (XCI) has hinted at a DNAme-independent mechanism responsible for the preferential inactivation of the paternal X in the extra-embryonic lineages of female mouse embryos (Takagi and Sasaki, 1975). Although earlier studies suggested that the maternally inherited allele of *Xist,* the lncRNA required for the initiation of XCI, is kept silent by DNAme directly inherited from oocytes (Ariel et al., 1995; Zuccotti and Monk, 1995), subsequent work with targeted or genome-wide bisulfite sequencing failed to confirm those results or reveal such a preemptive DNAme mark on the *Xist* promoter in eggs (McDonald et al., 1998; Shirane et al., 2013). Although the epigenetic imprint preventing silencing of the maternal X was shown to be established during oocyte growth, when DNAme marks are laid down (Tada et al., 2000), imprinted XCI was not perturbed in the progeny of *Dnmt3a/3b* mutant oocytes (Chiba et al., 2008), which fail to acquire DNAme (Hata et al., 2002; Kaneda et al., 2010; Shirane et al., 2013).

Together, these lines of evidence suggested that DNAme might not be the only epigenetic mark directly inherited from gametes that can lead to imprinted expression in the progeny. Note that all the evidence summarized above (for *Xist* and autosomal paternally expressed genes) pointed to a silencing mark inherited from the oocyte. The discovery of such a DNAme-independent mechanism, which has been called “noncanonical imprinting” (Inoue et al., 2017a), heralded new avenues of studies in genomic imprinting research in mammals. Features unique to noncanonical imprinting have been covered extensively by excellent recent reviews (Chen and Zhang, 2020; Hanna and Kelsey, 2021; Kobayashi, 2021; Albert and Greenberg, 2023; Inoue, 2023).

### 3.2 Noncanonical imprinting

The discovery of noncanonical imprinting emerged from elegant studies mapping allele-specific DNase I hypersensitive sites (DHSs) in zygotes and morulae. For these experiments, the group of Yi Zhang first established a low-input protocol for the genome-wide mapping of DHSs, liDNase-seq, suitable for preimplantation work (Lu et al., 2016). By individually analyzing the profiles of DHSs in the paternal and maternal pronuclei, they identified parental allele-specific DHSs priming allele-specific expression at the 2-cell stage (Inoue et al., 2017a). Allele-specific regions of open chromatin in early mouse embryos have also been independently mapped by ATAC-seq (Wu et al., 2016). Most of these open chromatin regions were of paternal origin and since the protection of the maternal allele at 48% of these sites did not overlap with DNA methylated regions in oocytes, the results provided support for a DNAme-independent mechanism silencing the maternal alleles (Inoue et al., 2017a). By mining ChIP-seq data for the Polycomb Repressive Complex 2 (PRC2)-mediated H3K27me3 marks in oocytes and by injection of the mRNA for the H3K27me3-specific demethylase KDM6B, Inoue et al. further showed that maternally inherited H3K27me3 was responsible for the observed protection of the maternal allele and for imprinted expression of those genes from the paternal allele in morulae (Inoue et al., 2017a). Subsequent similar studies from this group showed that the imprinted expression of the lncRNA *Xist*, responsible for paternal X chromosome inactivation in extraembryonic tissues, is also controlled via a similar noncanonical imprinting mechanism via maternal H3K27me3 marks (Inoue et al., 2017b). The genetic requirement for a functional PRC2 in the establishment of oocyte H3K27me3 imprints was shown in two independent studies documenting the loss of non-canonical imprinting at autosomal genes and *Xist* in the progeny of embryonic ectoderm development (*Eed*)- deficient oocytes (Inoue et al., 2018; Harris et al., 2019). The observation that imprinted expression is maintained at DNAme-dependent canonical imprinted genes in those *Eed* maternal KO progeny highlights the functional independence of both imprinting mechanisms (Inoue et al., 2018). This conclusion is also supported by the maintenance of noncanonical imprinted expression in the progeny of *Dnmt3l*-deficient females, confirming that noncanonical imprinting is independent of oocyte DNAme (Chen et al., 2019). In addition to the protection of maternal alleles from assuming an open chromatin state, the maternal H3K27me3 imprints also prevent the acquisition of activating H3K4me3 marks on the maternal allele in preimplantation embryos (Chen et al., 2019). Although the available data are consistent with H3K27me3 being the epigenetic mark directly inherited from oocyte, an interplay with the Polycomb Repressive Complex 1 (PRC1)-mediated H2AK119ub1 mark has also been described: whereas H2AK119ub1 coexists with and might precede H3K27me3 establishment during oocyte growth, its depletion in zygotes does not disrupts noncanonical imprinting, unlike what was seen for the H3K27me3 marks (Chen et al., 2021; Mei et al., 2021). Nevertheless, deletions of the PRC1.6 subunits PCGF1/6 in oocytes lead to partial loss of noncanonical imprinting genes in morulae (at 9/16 genes) (Mei et al., 2021).

The work summarised above revealed a DNAme-independent mechanism of imprinting, called noncanonical imprinting, however important differences with DNAme-dependent canonical imprinting were noted. Although more than 70 genes have been detected as noncanonically imprinted and paternally expressed in preimplantation embryos, all of these genes lose their imprinted expression in epiblast-derived post-implantation tissues (Inoue et al., 2017a; Santini et al., 2021). Nevertheless, maintenance of noncanonical imprinted expression has been observed in extra-embryonic tissues, including visceral endoderm at E6.5, extra-embryonic ectoderm (EXE) at E6.5 and E7.5, ectoplacental cone at 6 somite stage (∼E8.5), as well as in E9.5 and E12.5 placentae (Inoue et al., 2017a; Hanna et al., 2019; Andergassen et al., 2021; Zeng et al., 2021). Similar tissue-specific maintenance of noncanonical imprinting only in extra-embryonic lineages was observed via the mapping of allelic H3K4me3 promoter marks (Hanna et al., 2019). One exception is *Slc38a4*, which shows imprinted expression from the paternal allele in E13 fetus as well as tissue-specifically imprinted in adult adrenals, heart, and skeletal muscle (Smith et al., 2003). This is explained by the fact that *Slc38a4* is at least partially regulated by a gDMR, suggesting that both canonical and noncanonical mechanisms may regulate the expression of this gene in different tissues, perhaps via different isoforms (Smith et al., 2003; Inoue et al., 2017a; Bogutz et al., 2019; Chen and Zhang, 2020).

The expression data therefore suggest that noncanonical imprinting is mostly a transient mechanism, leading to paternal allele-specific imprinted expression of several genes (>70) in preimplantation embryos, but only maintained at some of these loci in extra-embryonic lineages (notably 7 genes: *Gab1, Phf17, Platr20, Sall1, Sfmbt2, Slc38a4,* and *Smoc1*). This would represent approximately 2.7% of mouse imprinted genes. The transient nature of this imprinting mechanism is consistent with the observation that the broad H3K27me3-marked regions inherited from the oocyte and required for noncanonical imprinting are largely maintained to the blastocyst stage, but are erased in E6.5 epiblast (Zheng et al., 2016), and are absent in embryonic stem cells, mouse embryonic fibroblasts, and adult somatic cells (Matoba et al., 2018). These observations provide an explanation for the biallelic expression seen for noncanonical imprinted genes in embryos generated by somatic cell nuclear transfer (cloning), since the maternal H3K27me3 marks responsible for noncanonical imprinting are absent in the somatic donor cells (Okae et al., 2014b; Matoba et al., 2018; Xie et al., 2022).

### 3.3 Evidence for LTR-guided imprint maintenance

Although noncanonical imprinting has been detected in post-implantation extra-embryonic lineages via expression and H3K4me3 data, the maternal H3K27me3 marks do not survive past the blastocyst stage (Chen et al., 2019; 2021). This raises the question of what are the mechanisms guiding and maintaining this maternal allele-specific silencing in extra-embryonic lineages of post-implantation embryos. By comparing the genomic localisations of paternal H3K4me3 peaks associated with imprinted expression, a key difference was noted between the two families of imprinted genes: at canonical imprinted genes, those H3K4me3 peaks are mostly associated with promoter CpG islands, while at noncanonical genes, the active promoter marks map to endogenous retroviral elements, notably of the ERVK family (Hanna et al., 2019). While these noncanonical imprinted ERVK promoters are not marked by H3K27me3 in E6.5 EXE, they are in fact marked by DNAme on the silent maternal allele. Since these DNAme marks at imprinted ERVKs are not present in preimplantation embryos, they constitute classical somatic DMRs (sDMRs) (John and Lefebvre, 2011), acquired in post-implantation embryos (Chen et al., 2019; Hanna et al., 2019). An essential role for both DNMT3A and DNMT3B in this postimplantation *de novo* methylation pathway was confirmed by ablating both genes in zygotes using CRISPR-Cas9. However, *Sfmbt2* appears to be an exception here, with its imprinted expression being maintained despite loss of DNAme, at least at E6.5 (Chen et al., 2019). Surprisingly, this effect at *Sfmbt2* was not observed in zygotic euchromatic histone lysine N-methyltransferase 2 (*Ehmt2*)-null embryos (also known as G9a), in which the establishment of the sDMRs at noncanonical imprinted genes does not occur and biallelic expression is observed (Auclair et al., 2016; Zeng et al., 2021).

**FIGURE 3:**
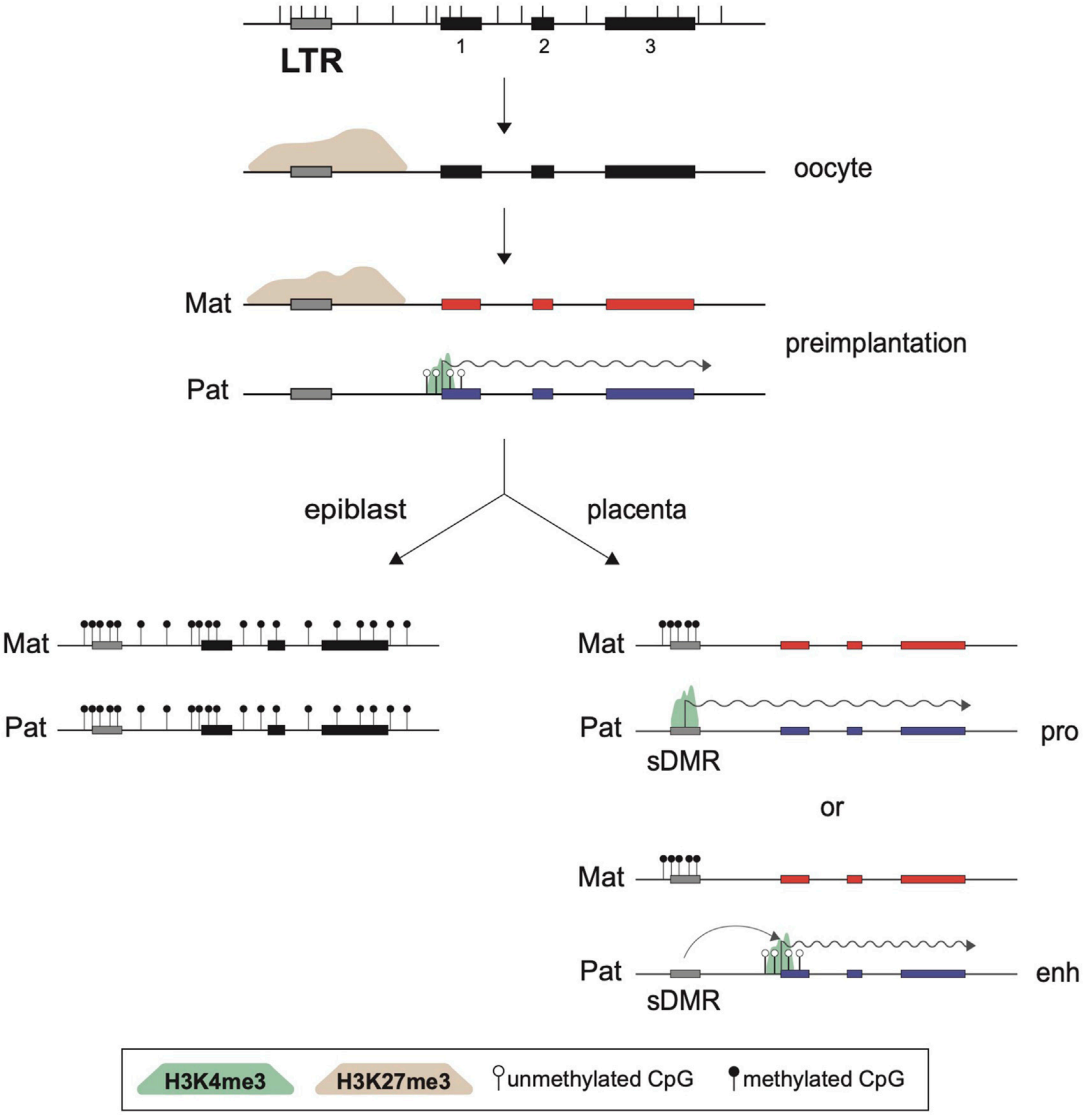
Role of LTRs in noncanonical imprinting maintenance. Structure of a noncanonical imprinted gene is presented at the top, showing three exons (black rectangles), the positions of CpG dinucleotides (vertical bars), and an upstream LTR element. In oocytes, part of the region is marked by a broad PRC2-deposited H3K27me3 domain (brown shade). This silencing epigenetic mark is inherited on the maternal allele such that only the paternal allele of the affected gene can be transcribed in preimplantation stages, as shown by the active H3K4me3 promoter mark (green shade) and transcription elongation (wiggly arrow). However, this histone imprint is only transient and is lost in all postimplantation cell lineages. In the epiblast, *de novo* DNA methylation leads to biallelic silencing, while in the extra-embryonic lineages a somatic DMR (sDMR) is generated over a nearby ERVK LTR element, with DNAme acquired exclusively on the previously H3K27me3-marked maternal allele. The LTR can then act as an alternative promoter (pro) or an enhancer (enh) to guide imprinted expression of the paternal allele. Not shown are the roles of PRC1 and its associated H2AK119ub mark in the establishment of the H3K27me3 domain in oocytes, or the implications of DNMT3A/3B, SMC hinge domain containing 1 (SMCHD1) and G9A/EHMT2 in formation of the extra-embryonic sDMRs themselves.

As expected, the sDMRs at imprinted ERVKs are also lost in the progeny of *Eed-*null oocytes, confirming the importance of the maternal H3K27me3 imprints in the initiation of this imprinting process. The observations that these ERVK elements become biallelically DNA methylated in the epiblast is consistent with the maintenance of noncanonical imprinting only in extra-embryonic lineages. Although some of these ERVKs, which are mostly solo LTR elements, were shown to act as alternative promoters for noncanonical imprinted genes, it remains to be seen whether some of these elements act as extra-embryonic enhancer elements, as has been previously reported for some LTR families (Chuong et al., 2013; Hanna et al., 2019; Figure 3). How the sDMRs are established specifically in extra-embryonic lineages but not in the epiblast-derived tissues is also currently unknown.

Together, this body of work has revealed that noncanonical imprinted genes, which so far have only been observed to be paternally expressed in extra-embryonic tissues, may rely on alternating allelic epigenetic marks for their allelic expression. The oocyte-derived H3K27me3 imprint, itself dependent on H2AK119ub1 at certain genes, must be converted into a DNAme somatic mark only on the maternal allele to achieve noncanonical imprinting in extra-embryonic tissues. This switch may be guided by the paternal H3K4me3 promoter marks over ERVK elements in preimplantation embryos, which would protect the paternal alleles from the action of the DNMT3A/3B *de novo* enzymes (Zhang et al., 2010). The observation that both the paternal H3K4me3 peaks and the sDMRs implicated in noncanonical imprinting map to endogenous retroviral promoters, notably of the ERVK family, suggests that these elements play critical roles in the maintenance of this unique tissue-specific imprinting pathway. In the cases where the imprinted ERVK element act as an alternative promoter for paternal allele-specific expression, a parallel can be drawn with the role of LTRs in the establishment of canonical imprinting, for which their activity as an oocyte promoter is critical.

## 4 Concluding remarks

Canonical imprints are essential for embryonic development, as shown by the early midgestational lethality of offspring obtained from oocytes deficient in *de novo* DNAme (Bourc’his et al., 2001; Hata et al., 2002; Kaneda et al., 2004). Although much remains to be determined regarding the biological functions of noncanonical imprinted genes in extraembryonic lineages, the global loss of maternal H3K27me3 marks in conditional *Eed* or *Ezh2* mutant oocytes is compatible with development to term in the progeny, although with embryonic growth defects (Erhardt et al., 2003; Prokopuk et al., 2018). The shared placental overgrowth phenotypes observed in cloned mice and in *Eed* maternal KO conceptuses have been linked to the abnormal expression of noncanonical imprinted genes such as *Slc38a4, Gm32885* (a transcript upstream of *Slc38a4* on Chr 15)*,* as well as a cluster of microRNAs coded within an intron of *Sfmbt2,* C2MC (Matoba et al., 2018; 2019; 2022). Together, these observations support key developmental roles for imprinted genes, both canonical and noncanonical, with an emphasis on the regulation of extra-embryonic lineages.

The fact that LTR elements, which are highly polymorphic in different mammalian species, are implicated in different aspects of both imprinting pathways suggests that they may play important roles in the emergence of new imprinted genes in different species. In their function as oocyte promoters for the establishment of maternal DNAme marks, LTRs were shown to be involved in the imprinting of non-overlapping sets of canonical imprinting genes in mouse and human (Bogutz et al., 2019). However, the three protein-coding genes imprinted by an oocyte LTR promoter in mouse (*Slc38a4, Impact,* and *Chd15*) are also imprinted in rat (Albert et al., 2023). A different picture emerges for noncanonical imprinted genes: although several noncanonical imprinted genes identified in mouse appear conserved in rat, profiling of allelic usage in this species also identified 8 rat-specific putative noncanonical imprinted genes, consistent with a rapid evolution of this imprinting mechanism in rodents (Albert et al., 2023). Nevertheless, whether the noncanonical pathway also operates in human embryos is unclear, since human *XIST* expression is not imprinted in preimplantation embryos nor in extra-embryonic membranes (Migeon and Do, 1979; Petropoulos et al., 2016), and most H3K27me3 marks are rapidly erased in human preimplantation embryo (Zheng et al., 2016; Xia et al., 2019; Lu et al., 2021). On the other hand, other studies have reported maternal-biased H3K27me3 marks and associated paternal allele-specific expression in human morulae (Zhang et al., 2019), as well as the presence of placental sDMRs corresponding to regions marked by H3K27me3 and hypomethylated in eggs, which are consistent with putative noncanonical imprinting (Hanna and Kelsey, 2021). Future work on other mammalian species will be important to establish the conservation and importance of LTR-based mechanisms of imprinting in the evolution of new imprinted genes.
